# Xanthohumol microbiome and signature in healthy adults (the XMaS trial): a phase I triple-masked, placebo-controlled clinical trial

**DOI:** 10.1186/s13063-020-04769-2

**Published:** 2020-10-07

**Authors:** Ryan Bradley, Blake O. Langley, Jennifer J. Ryan, John Phipps, Douglas A. Hanes, Emily Stack, Janet K. Jansson, Thomas O. Metz, Jan Frederik Stevens

**Affiliations:** 1 National University of Natural Medicine, Portland, USA; 2 Pacific Northwest National Laboratory, Richland, USA; 3 Oregon State University, Corvallis, USA

**Keywords:** RCT, Human, Safety, Microbiome, Xanthohumol, Inflammation

## Abstract

**Background:**

Natural products may provide a source for the discovery and development of adjunctive pharmacological interventions to modulate the inflammatory pathways contributing to chronic disease. Xanthohumol, a flavonoid from the hops plant (*Humulus lupulus*), has antioxidant and anti-inflammatory properties and may act as a prebiotic to the intestinal microbiota. Xanthohumol is not currently approved as a drug by the US Food and Drug Administration (FDA), but is available as a dietary supplement and ingredient in medical foods. To formally test the safety of xanthohumol, a phase I clinical trial (“XMaS”) was designed and approved under an Investigational New Drug application to the US FDA. The main objective is to examine the clinical safety and subjective tolerability of xanthohumol in healthy adults compared to placebo. Additional aims are to monitor biomarkers related to inflammation, gut permeability, bile acid metabolism, routes, and in vivo products of xanthohumol metabolism, and to evaluate xanthohumol’s impact on gut microbial composition.

**Methods:**

The safety and tolerability of xanthohumol in healthy adults will be evaluated in a triple-masked, randomized, placebo-controlled trial. Participants will be randomized to either 24 mg/day of xanthohumol or placebo for 8 weeks. Blood cell counts, hepatic and renal function tests, electrolytes, and self-reported health-related quality of life measures will be collected every 2 weeks. Participants will be queried for adverse events throughout the trial. Xanthohumol metabolites in blood, urine, and stool will be measured. Biomarkers to be evaluated include plasma tumor necrosis factor-alpha, various interleukins, soluble CD14, lipopolysaccharide-binding protein, fecal calprotectin, and bile acids to assess impact on inflammatory and gut permeability-related mechanisms in vivo. Stool samples will be analyzed to determine effects on the gut microbiome.

**Discussion:**

This phase I clinical trial of xanthohumol will assess safety and tolerability in healthy adults, collect extensive biomarker data for assessment of potential mechanism(s), and provide comparison data necessary for future phase II trials in chronic disease(s). The design and robustness of the planned safety and mechanistic evaluations planned provide a model for drug discovery pursuits from natural products.

**Trial registration:**

ClinicalTrials.gov NCT03735420. Registered on November 8, 2018

## Administrative information

**Table:** 

**Title {1}**	*Xanthohumol Microbiome and Signature in Healthy Adults (the XMaS Trial): A Phase I Triple-masked, Placebo-controlled Clinical Trial*
**Trial registration {2a and 2b}.**	ClinicalTrials.gov (NCT03735420) and posted on November 8th, 2018 All items per WHO trial registration requirements can be found within the body of the protocol.
**Protocol version {3}**	Version 1.8 11/21/2019
**Funding {4}**	• National Center for Complementary and Integrative Health (NCCIH): Grants 1RO1AT010271-01, R01 AT010271-02S1 • Hopsteiner, Inc.: XanthoFlav Pure product • Metagenics, Inc.: Xanthohumol and placebo capsules
**Author details {5a}**	Drs. Bradley, Stevens, Ryan, and Phipps have previously received grant support and study product donations from Metagenics, Inc. which supported the phase I XMaS study by encapsulating the test materials used in this trial.
**Name and contact information for the trial sponsor {5b}**	Yisong Wang, Program Director, NCCIH yisong.wang@nih.gov
**Role of sponsor {5c}**	This trial is an investigator-initiated and NUNM-sponsored clinical trial funded by the NCCIH of the National Institutes of Health (NIH). The NIH and associated reviewers provided feedback on trial participation criteria which were incorporated into the trial protocol, as was necessary to secure NIH approval to recruit. The NIH did not influence the conduct, analysis, writing or decision to submit the protocol for publication. Although encapsulation of the study product was provided by Metagenics, Inc., employees of Metagenics, Inc. did not have any involvement in the study design, collection of data, or manuscript development. Funders will not be involved in data analysis, interpretation, or future manuscript development. None of the authors hold stock in Metagenics, Inc. or its parent company and have no direct financial ties to the outcome of the proposed clinical research.
**Submission Deadline**	This trial has not reached maximum enrollment to date, March 24, 2020. Although the trial is nearing the final stages prior to completion, there was insufficient time for the study team to complete a protocol manuscript until Dr. Langley was fully trained on and incorporated into the trial’s operations. We respectfully submit this protocol manuscript on March 24, 2020.

## Introduction

### Background and rationale {6a}

Xanthohumol is a flavonoid constituent of the hops plant (*Humulus lupulus*), exerting antioxidant, anti-inflammatory, cancer chemopreventive, anti-hyperglycemic, and anti-hyperlipidemic activities [1–3]. Xanthohumol also acts as a prebiotic for intestinal microbiota and can alter the gut microbial composition in conjunction with its bacterial metabolites [4, 5]. Based on mechanisms of action supported by preclinical models, patients with numerous clinical conditions could potentially benefit from xanthohumol supplementation (Table 1). These effects come from known influences of xanthohumol on transcription factors that may provide upstream effects on inflammatory processes [6]. For instance, transcription factors that regulate gene expression known to be involved in the pathogenesis and progression of a broad array of human diseases are modulated by xanthohumol, including nuclear factor-kappa B (NFκB) [7–10], nuclear factor erythroid 2-related factor 2 (NRF2) [11, 12], and the farnesoid X receptor (FXR) [13, 14] (see Table 1).

**Table 1:** Biologic mechanisms of xanthohumol on transcription factors and pertinent clinical conditions

Transcription factor	Xanthohumol mechanisms	Pertinent clinical conditions
Farnesoid X receptor (FXR)	FXR agonist in vitro and in vivo	• Type 2 diabetes mellitus • Metabolic syndrome • Dyslipidemia • Obesity • Non-alcoholic fatty liver disease • Non-alcoholic steato-hepatitis • Cholelithiasis • Inflammatory bowel disease • Cancer
Nuclear factor-kappa B (NFkB)	Inhibits activation of NFkB in vitro and in vivo	• Inflammatory bowel disease • Rheumatic disease • Asthma • Autoimmunity • Cancer
Nuclear factor erythroid 2-related factor 2 (NRF2)	Increases expression of and activation of NRF2 in vitro	• Autoimmunity • Alzheimer’s disease • Inflammatory bowel disease • Cancer

Using in vitro binding studies and in silico models, xanthohumol and its metabolites are ligands for the FXR and preliminary in vitro data indicates xanthohumol activates FXR-regulated genes at low concentrations, supporting xanthohumol functions as a pharmacologic agonist of FXR [4, 13]. The FXR receptor is known to regulate intestinal permeability as well as innate immunity including production of the pro-inflammatory cytokines: interleukin (IL)-1β, IL-6, IL-8, IL-10, IL-12p70, tumor necrosis factor alpha (TNF-α), and interferon-gamma (IFN-γ) [15, 16]. FXR agonists have been tested in animal models and early clinical trials, showing promise for altering inflammatory signaling pathways [15, 17]. Thus, xanthohumol has the potential to be a novel therapeutic in pertinent diseases (e.g., inflammatory bowel disease) by acting as an FXR agonist, reducing inflammation, modulating bile acid metabolism, and reducing gut permeability. However, xanthohumol is not currently approved as drug by the FDA for the treatment, cure, prevention, or mitigation of disease, despite being available as a dietary supplement in both crude and standardized extracts from hops.

For a segment of the population, xanthohumol is commonly consumed in the diet through beer, which contains 0.5–4 mg/L depending on brewing processes [18]. Thus, xanthohumol appears safe when administered orally in amounts commonly contained in the diet through beer. Furthermore, both hops and hops oil are considered Generally Recognized as Safe in the USA [19]. Animal models have shown body morphological changes, including decreased weight in some models, without effect on reproduction or histopathological changes in the bone marrow, liver, exocrine pancreas, kidneys, muscle, thyroid, ovaries, and testes or changes in complete blood counts in doses up to 1000 mg/kg of body weight [20–22]. Although xanthohumol has been formally evaluated in a pharmacokinetics study in humans, no previous studies have prospectively evaluated the safety and tolerability of xanthohumol, as an isolated constituent, in human subjects [23].

In the planned phase I XMaS trial, healthy participants will be recruited and randomized to either 24 mg of xanthohumol in a rice protein vehicle or rice protein placebo for 8 weeks. Routine clinical biomarkers and quality of life of the participants will be evaluated in 2-week intervals to examine the safety and tolerability of oral supplementation with xanthohumol. Additional aims are to collect data on biomarkers related to inflammation, gut permeability, bile acid metabolism, routes of xanthohumol metabolism, and potential impacts on gut microbiota.

## Methods/design

### Objectives {7}

The primary aim of this trial is to determine the safety and tolerability of xanthohumol in healthy adults through administration of 24 mg/day of xanthohumol, compared to placebo. This aim will be met by assessing routine clinical toxicology measures compared to baseline values, including complete blood count, electrolytes, estimated glomerular filtration rate, blood urea nitrogen to creatinine ratio, aspartate aminotransferase (AST), alanine aminotransferase (ALT), ɣ-glutamyl transferase (GGT), alkaline phosphatase, and bilirubin, plus self-reported adverse events. The secondary aim is to measure effects of xanthohumol on TNF-α as a biomarker of active inflammation [14, 16, 24, 25]. The tertiary aim is to collect preliminary data related to effects on gut permeability, xanthohumol metabolism, and impact on the gut microbiome for a subsequent phase II trial in adults with Crohn’s disease based on mechanisms described in Table 1. Tertiary outcome measures include xanthohumol metabolites in the blood and urine; biomarkers of gut permeability, enterocyte inflammation, and systemic immune activity; assessment of bile acid metabolism; gut microbial composition; and identification of gut microbial proteins targeted by xanthohumol. Specific biomarker information by aim can be found in Table 2. Summary safety data will be reported upon trial completion as the frequency of new-onset laboratory abnormalities, including changes in anemia status, liver function tests (AST, ALT, GGT, bilirubin, and alkaline phosphatase), electrolytes, and/or creatinine and estimated glomerular filtration rate (eGFR).

**Table 2:** Aims and methods of assessment

Aim	Marker	Assessment method
1. *Safety & Tolerability*	a. RBC, WBC, platelets b. eGFR, electrolytes, BUN:Cr, AST, ALT, GGT, alkaline phosphatase, bilirubin c. Adverse events, quality of life	a. Complete Blood Count (CBC) b. Comprehensive Metabolic Panel (CMP) c. Adverse Events Questionnaire, PROMIS-29
2. *Inflammation*	a. Inflammatory cytokines: IL-1β, IL-2, IL-6, IL-8, IL-10, TNF-α, and IL-12p70 b. Fecal calprotectin	a. Serum or plasma analysis b. Stool analysis
3a. *Gut Permeability*	a. CD14, lipopolysaccharide binding protein, intestinal fatty acid binding protein	a. Plasma analysis
3b. *Xanthohumol Metabolism*	a. Xanthohumol metabolite profiles	a. 24-hour urinalysis, plasma analysis, stool analysis
3c. *Microbial Composition Changes*	a. Metagenomic DNA sequencing and taxonomic profiling b. Proteomic characterization	a. Compared to xanthohumol metabolite profiles b. Activity-based proteomic assay

## Hypotheses

We hypothesize that xanthohumol, at a dosage of 24 mg per day, will be safe and tolerable in trial participants as demonstrated by frequency of laboratory abnormalities or reported adverse events observed between groups not being significantly higher, and at most 50% higher in the intervention group. Additionally, we hypothesize xanthohumol and its microbiota-derived metabolites will reduce biomarkers of inflammation and modify barrier function in the inflamed gut. We anticipate xanthohumol will produce a specific microbiota-based biological mechanistic signature, which will provide the groundwork to determine its therapeutic potential in various chronic diseases.

### Trial design {8}

This study is a phase I, two-arm, 1:1, randomized, triple-masked, placebo-controlled clinical trial.

## Materials and methods

### Study setting {9}

All clinical visits, including screening and informed consent, will be conducted in an academic clinic at the Helfgott Research Institute in Portland, OR.

### Eligibility criteria {10}

**Fig. 1:**
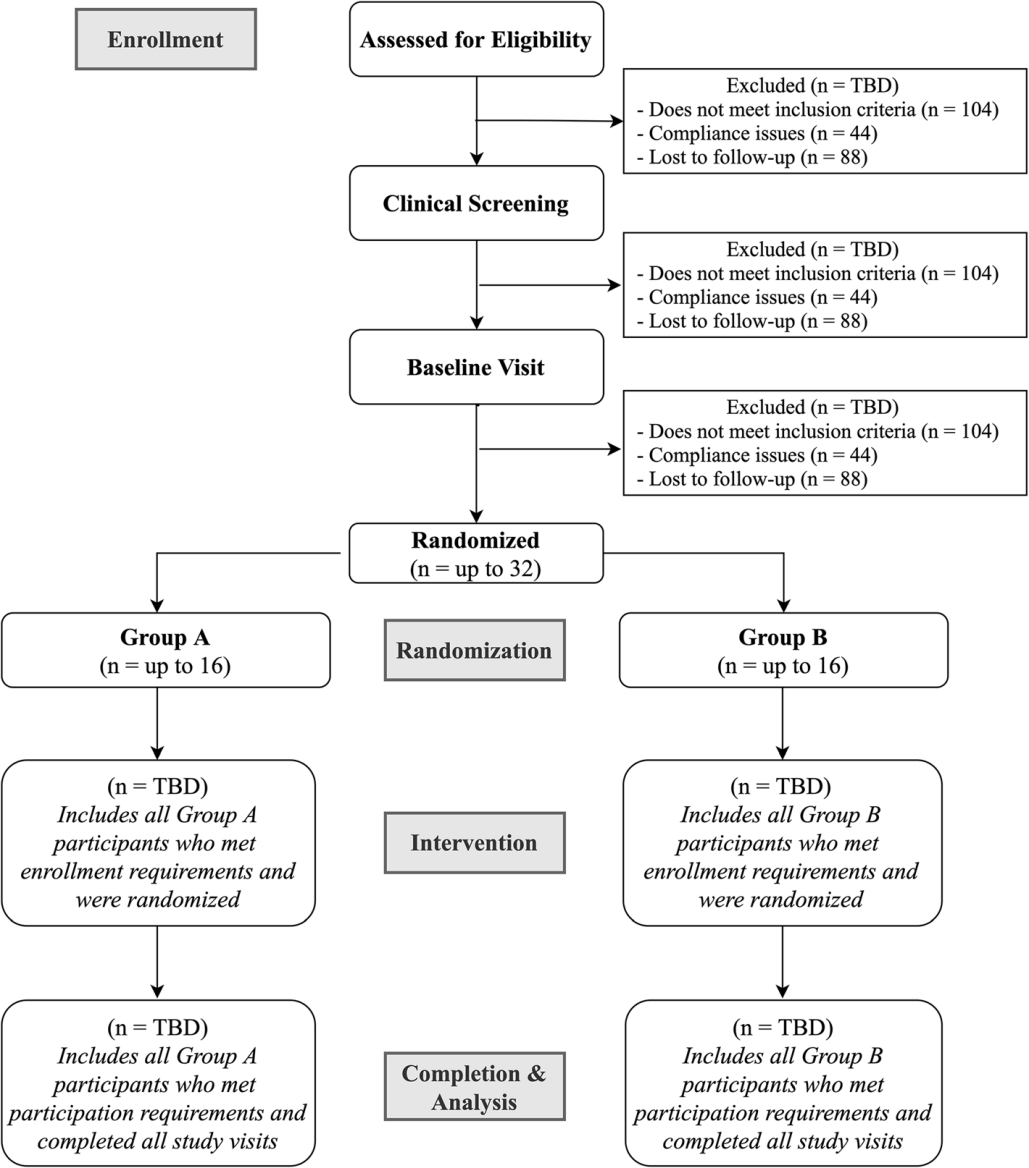
Enrollment and allocation

**Fig. 2:**
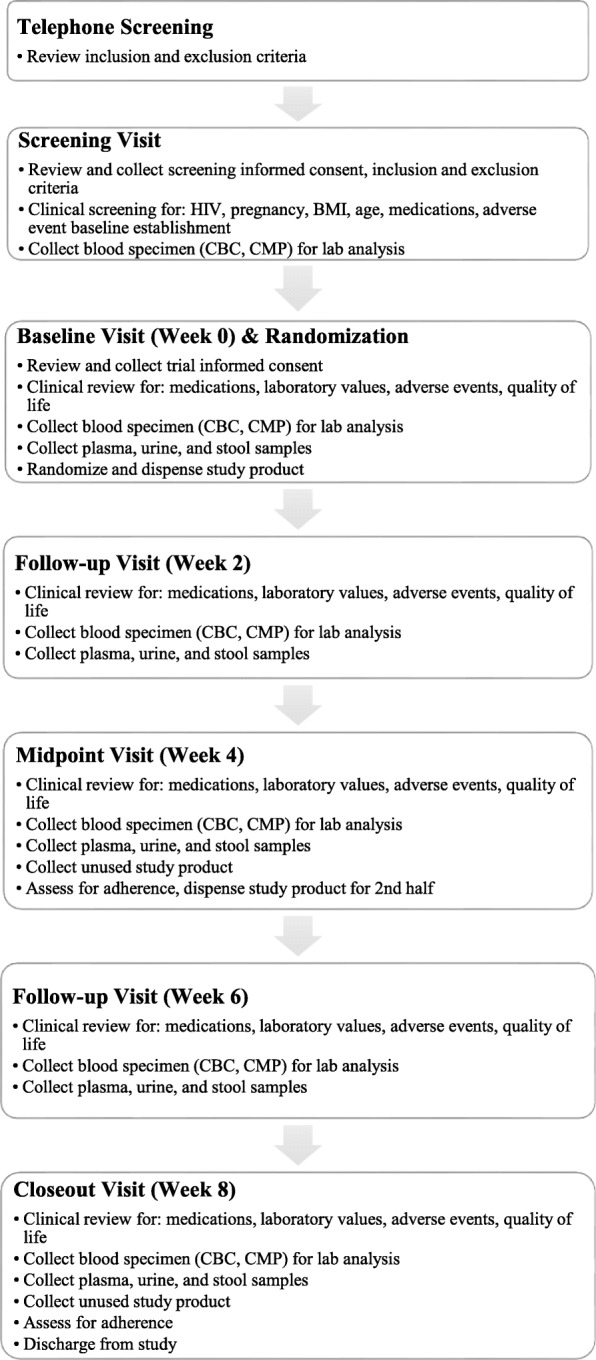
Flow of study procedures

Eligibility criteria can be found in Table 4. Diagrammatic and tabular explanations of the overall trial design, flow of study procedures, and detailed study visit descriptions can be found in Figs. 1 and 2 and Table 3, respectively.

**Table 3:** Criteria for inclusion and exclusion

Inclusion criteria	Exclusion criteria
• Males and females aged 21–50 years • BMI 20–30 • Willing to be randomized to take isolated xanthohumol as a dietary supplement (or placebo) for 8 weeks • Willing to have blood drawn every 2 weeks and fast for 10–12 h before blood draws • Willing and able to collect stool samples at home every 2 weeks • Able to speak, read, and understand English • Must be able to provide written informed consent • Non-smokers (including tobacco and cannabis products, combusted or vaporized) *Permitted concomitant care or intervention during the trial:* • Use of over-the-counter medications as indicated on the label • Multi-vitamin/multi-mineral products • Dietary supplements except as detailed in exclusion criteria	• History of any chronic disease • Consumption of more than 1 microbrew beer per day • Typical intake of more than 2 alcohol-containing beverages per day, more than 14 per week, or more than 4 in any single day within 28 days prior to screening • Use of NSAIDs more than once per week for headaches, routine aches/pains, etc. • Use of any prescription drugs, including oral contraceptives (due to potential interference with mechanisms under investigation) • Acute viral or bacterial infection, or recent infection within 14 days prior to screening or still requiring prescription medication for treatment • Engaging in vigorous exercise more than 6 h per week • Women who are lactating, pregnant, or planning pregnancy during the study period • Within 3 months of screening: ° Use of prescription opioids for any reason ° Use of prescription corticosteroids for any reason ° Hospitalization (for any reason other than an elective medical procedure) ° Gastrointestinal surgery • Current or within 30 days of screening: ° Intravenous nutrition support therapy ° Intake of anti-coagulant or anti-platelet prescription medications ° Intake of antibiotic, antiparasitic, or antifungal medications orally or intravenously ° Initiation of or changes to supplements or medications, an exercise regimen, or food plan ° Involvement in a significant diet or weight loss program, low-carb diet program, or very-low calorie liquid diet program ° Use of recreational drugs/substances ° Participation in another interventional research study ° Undergoing UV therapy • Within 14 days of screening: ° Recent acute trauma occurring within 14 days prior to screening ° Recent (within 14 days prior to screening) intake of any dietary supplements containing xanthohumol flavonoids, or other natural products typically taken to modulate inflammation including curcumin, turmeric, fenugreek, hops, rosemary, ginger, white willow, devil’s claw, fish oil (doses > 1 g/day), bioflavonoids, or quercetin

### Screening and informed consent {26a, 26b}, confidentiality {27}

Measures to ensure the privacy of information on study participants will be maintained throughout the trial. All study investigators and staff are certified in Good Clinical Practices, Human Subjects Research, and Responsible Conduct of Research and have received training in clinical HIPAA regulations. Telephone screening will be conducted via verbal interview to assess inclusion and exclusion criteria noted in Table 4. Eligible participants will then be invited to an in-person, clinical screening visit. Screening informed consent will be collected from all participants at the screening clinical visit and cover collection and storage of medical history as well as collection and analysis of biological specimens. Trial informed consent will be collected from all participants at the baseline clinical visit and will review the collection and analysis of biological specimens, the option to supply additional samples to be stored for future research purposes, and all other trial operations.

**Table 4:**
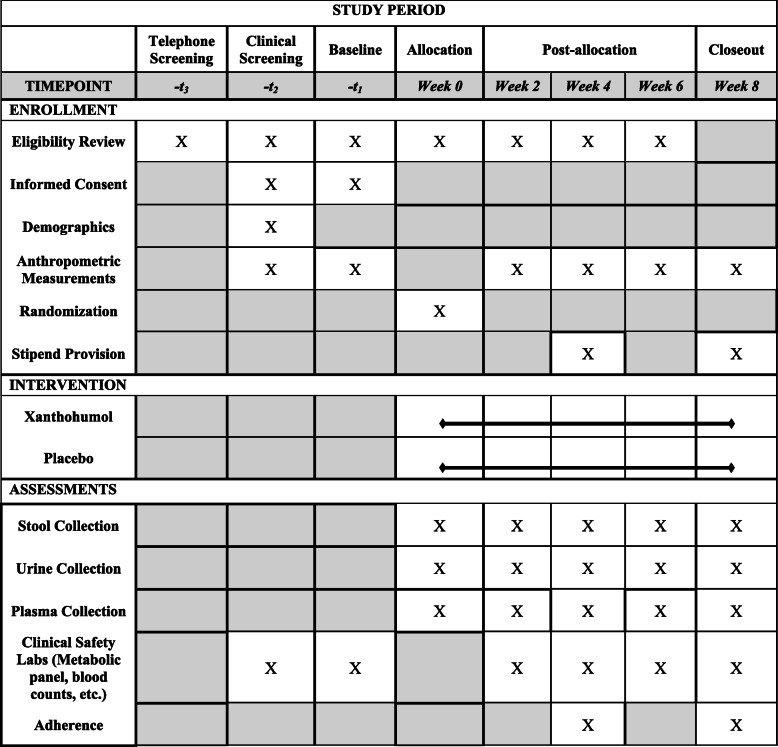
Participant timeline

## Interventions

### Intervention description {11a} and choice of comparators {6b}

Participants randomized to xanthohumol will be administered 24 mg of 99+% pure xanthohumol combined with 288 mg of rice protein, 109.3 mg microcrystalline cellulose, 4.3 mg Aerosil® 200 fumed silica, and 4.3 mg magnesium stearate, in an orange capsule to maintain masking. Xanthohumol has been combined with a rice protein carrier as this has been shown to significantly improve xanthohumol bioavailability in human subjects [26]. Participants randomized to placebo will be administered 288 mg of rice protein, 133.4 mg microcrystalline cellulose, 4.3 mg silica, and 4.3 mg magnesium stearate combined in an identical capsule. Xanthohumol is considered odorless and the capsules produce no residue.

### Criteria for discontinuing or modifying allocated interventions {11b, 21b}

A participant may be withdrawn from the trial at any time they choose, due to adverse events or at their request. However, participants will be actively withdrawn if they demonstrate any new onset, moderate, or more severe adverse events attributable to the intervention. Participants will also be withdrawn if they have any critical value return on routine clinical laboratory measures of safety, including significant changes in blood cell counts, liver function tests, or renal function tests. The trial will be suspended if > 20% of study participants experience moderate or greater severity adverse events or laboratory abnormalities contributing to halting criteria including abnormal calcium, sodium, potassium, creatinine, or GFR; anemia; and/or abnormal elevations in bilirubin, elevated alkaline phosphatase, AST, or ALT that are persistent upon repeat testing.

### Adherence to intervention {11c}

Data on adherence to the treatment protocol will be assessed by study product return and pill counts at the week 4 and week 8 clinical visits. Participants will be informed that the returned study product will be counted to determine adherence to the protocol. Each visit will include verbal queries regarding any missed doses, extra doses, or lost capsules in order to be able to account for all study product. Study coordinators are trained to encourage use of reminder alarms and/or other tips to encourage adherence, as needed. Taking at least 80% of the required doses will be considered adherent.

### Relevant concomitant care permitted or prohibited during the trial {11d}

See Table 3.

### Provisions for post-trial care {30}

All randomized study participants who take the study product will be instructed to report adverse events to the trial investigators for 30 days following their discharge from the trial. Any new-onset adverse event not established at screening or during the trial, whether reported spontaneously or at the closeout clinical visit, will be followed up on via telephone by the study coordinator to determine its outcome. Throughout the study, if laboratory values, vital sign measurements, or self-reported adverse events arise, the study team will expediently make referrals to a primary care provider or to mental health crisis resources in Portland, OR. Because the experimental drug contains only a compound considered Generally Recognized as Safe, and is readily available in the diet, no serious harms are anticipated upon discontinuation of the drug that would require medical intervention.

### Participant timeline {13} and data management {19}

All clinical visits, including screening and informed consent, will be conducted at the Helfgott Research Institute in Portland, OR. Verbal interviews will be repeated to confirm participation criteria are met. During the clinical screening visit, salivary HIV screening and urinary pregnancy tests will be performed by study staff, as well as a series of anthropometric measurements, vital signs, medication usage, blood samples, and recent adverse event history for later comparison as stated in Fig. 2 and Table 3.

Data collected from clinical screening visits and throughout the study period will be maintained as both paper case report forms (CRFs) and electronic copies entered into a Research Electronic Data Capture (REDCap) [27] database in a de-identified manner on a password-protected tablet by study staff. During baseline visits, study staff will verbally administer questionnaires for adverse events, collect vital signs and anthropometric values, and administer the Patient-Reported Outcomes Measurement Information System (PROMIS-29) questionnaire which measures health-related quality of life across disease categories [28]. Data entered for adverse events and PROMIS-29 will be considered final data as the study team member conducting the visit will be entering those results directly into the REDCap database. All other data initially recorded into REDCap will be marked as “unverified” within the system to later be confirmed by a member of the study team, additionally regulated in REDCap by implementing specified allowable ranges of input to minimize the chance of data entry errors, as systems of quality control. Additional methods for ensuring confidentiality and data protection are outlined below.

For every clinical visit, specimen collection including 24-h urine and stool collection will occur as outlined in Table 4. During their time enrolled in the study, participants will be expected to take one capsule of the assigned product daily and record of any emerging symptoms to be collected at their next clinical visit. All adverse event reports will be collected through case report forms (CRFs) and documented in REDCap database for Data Safety Monitoring Board (DSMB) monitoring and post-trial analysis.

### Sample size {14}

A sample size of twenty-four (*n* = 12 per group) provides 80% power to detect a 50% difference in proportions of participants between groups by chi-squared test experiencing a laboratory abnormality or adverse event at a significance threshold of *α* = 0.05. To account for up to 25% attrition, up to 16 adults (8 biologically male and 8 biologically female participants) will be enrolled in each study arm. Given that this phase I trial is intended as a step toward a phase II clinical trial in Crohn’s disease, for which few effective treatments exist, the investigational drug is commonly available in the diet, and previous human studies have not suggested any toxicity, the present trial was powered to detect gross toxicity resulting in frequent adverse events and/or laboratory-measurable toxicity. However, interim halting criteria were also used to assess safety; an accumulated frequency of 25% reaching laboratory halting criteria was pre-specified as a criterion for halting the trial and requiring detailed review by the FDA, NCCIH, and IRB.

### Recruitment {15}

Participants will be recruited for the trial via flyers, word-of-mouth recommendation, and online advertisements in sources local to the Portland, OR metro area. Retention strategies include the use of stipends dispensed for clinical screening and each clinical visit completed following enrollment. The study aims to recruit a sample that is 50% male and 50% female in order to maximize the generalizability of our findings to both men and women.

## Assignment of interventions: allocation

### Sequence generation {16a}, concealment mechanism {16b}, implementation {16c}, blinding {17a}, and unblinding {17b}

Randomization will occur within 30 days of screening by blocking within the randomization sequence, with randomization stratified by biological sex. The initial randomization series will be generated using readily available electronic random sequence generators. The randomization “code” will be created by a staff member not involved in trial operations and kept in a sealed envelope with a signature across the label and dated the day of creation. This envelope will be kept in a dedicated study folder and locked in the office of the Research Integrity Officer or designee. Thirty-two opaque envelopes will be created, sequentially numbered include a designation of the code for the assigned intervention based on the randomization sequence, and signed across the seal. Masking of the interventions (i.e., xanthohumol or placebo) will be accomplished by engaging trained staff not involved in trial operations to label product bottles based on the randomization code. Product bottles will be stored in separate locked cabinets by code and maintained in a temperature-controlled environment, with temperature logged weekly to assess environmental stability. Upon confirmation of eligibility and consent, Study Coordinators will then open the next sequentially numbered envelope at the time of randomization, the envelope number will be recorded, and allocation (As “A” or “B”) disclosed to both participant and coordinator for the first time, and the corresponding product will be removed from its locker and dispensed in the bottle to the participant. Thus, the trial will be triple-masked without PIs, co-investigators, study coordinators, or participants knowing allocation assignment of study participants. Unmasking is permissible if the DSMB requires it upon review and in the case of a serious adverse event.

## Data collection, management, and analysis

### Plans for assessment and collection of outcomes {18a}

Each participant will be assigned a unique alpha-numeric ID upon screening. All blood and urine aliquot tubes, stool collection vials, and associated paperwork will be marked using the unique ID to protect participant confidentiality. All data resulting from study visits will be collected on standardized CRFs and through administered survey data. Data from CRFs will be transferred to a secure REDCap database, with survey entry entered directly, for data management and subsequent quality control checks [27]. Periodic data audits will be conducted monthly throughout the trial, and all data in REDCap will be confirmed from source documents prior to any analyses. All data will be kept for 3 years following completion of all grant activities. All source documents are identified only by an alphanumeric study ID and stored in a locked filing cabinet in a locked document storage room; all electronic data are stored on REDCap on a secure server requiring separate passwords to access the server and REDCap. REDCap user rights are carefully controlled to only allow the biostatistician, coordinator, and PI to export study data, all of which are de-identified.

### Plans to promote participant retention and complete follow-up {18b}

Retention will be promoted by a determined monetary compensation strategy with disbursement of funding after week 4 and week 8 clinical visits. For situations in which the participant is unable to complete an in-person clinical visit, protocol deviations will be followed on each case-by-case basis upon approval by the investigation team.

### Plans for collection, laboratory evaluation, and storage of biological specimens for genetic or molecular analysis in this trial/future use {33}

Blood-based specimens collected from study participants for clinical safety parameters will have an orchestrated same-day delivery to a Quest Diagnostics lab for analysis. Other blood-based samples for plasma analysis will be centrifuged at 2200–2500 rpm for at least 15 min prior to serial 500 μL aliquots to be stored in < − 70 °C at the study site. Urine specimen collected will be measured for overall collected volume, and serial 1 mL aliquots will be stored in < −70 °C at the study site. Stool specimens collected will be stored according to the specific requirements of the sample type: room temperature for microbial DNA analysis and < −70 °C for all other sample types at the study site. Samples stored at the study site will be labeled for identification using a format including study ID, visit number, and date collected until ready for analysis by partner sites upon which time transportation will be arranged to ensure sample integrity.

Study participants may indicate willingness for the study team to collect and store additional biological specimens at the baseline clinical visit and provide verbal confirmation at each subsequent clinical visit. These specimens will include up to four 1-mL vials each of urine and plasma for future analysis with respect to xanthohumol.

### Laboratory analysis outcomes {12}, statistical methods {20a, 20b, 20c}

Relevant laboratory-based safety measures were chosen as the primary safety outcomes, because this trial is intended to precede a clinical trial in adults with Crohn’s disease—a clinical population potentially vulnerable to anemia, liver toxicity, renal abnormalities, etc. Therefore, a formal assessment of laboratory changes in healthy humans provides critical information to guide expectations for changes in laboratory markers and associated adverse clinical effects in future research in Crohn’s and other clinical populations.

The primary objective’s laboratory-based outcome domains (and corresponding measures) are liver function (AST, ALT, alkaline phosphatase, bilirubin), renal function (BUN, creatinine, estimated glomerular filtration rate), hematology (red blood cell count, hematocrit, hemoglobin, white blood cell count), and electrolytes (sodium, potassium, calcium, chloride) measured every 2 weeks for 8 weeks. The primary outcome metric for comparison between study groups is the mean change from baseline to week 8. However, all parameters will be similarly evaluated at each follow-up time point, by formally comparing mean change from baseline to follow-up between groups. Comparisons at a single follow-up will use 2-sided independent *t* tests; omnibus tests for differing trends over time will employ linear mixed models with time point as a repeated factor and study group as an independent factor.

Primary outcome laboratory measures will be analyzed by first assessing descriptive statistics including mean, median, and standard deviation. All distributions will be tested for skew and transformed (e.g., natural log transformed) as needed to reduce the influence of skew in subsequent analyses. If inspection of distributions indicates significant non-normality that cannot be corrected with standard transformations, significance will instead be calculated using a non-parametric Wilcoxon signed rank test. The primary analysis will be a per-protocol analysis as this trial is a phase I trial focused on clinical toxicity, not a definitive efficacy or effectiveness trial; therefore, we are investigating the clinical and mechanistic effects of the product for those participants who took the experimental agent only. As a sensitivity analysis, missing laboratory data will be imputed by multiple imputation to assess the robustness of study results to imputation.

As an element of the evaluation for safety of the intervention, the percentage of laboratory newly abnormal (for values that were within the clinically normal range at baseline) per clinical laboratory reference ranges for each lab parameter will be reported at each time point and the percentage will be compared between groups by Fisher’s exact test. Assessment for changes in the distribution of each lab parameter will be performed by presenting means and confidence intervals (CI) for the change in each group, as well as the mean CI for the difference between groups; significance will be tested using 2-sided, unpaired *t* tests (or, if inspection of distributions indicates strong non-normality, with a non-parametric Wilcoxon signed rank test) of the mean values for the xanthohumol group compared to the placebo group. Assessment for overall *increases* or *decreases* in collected parameters will be tested for linear trends of the means by linear mixed ANOVA, considered significant if *p* < 0.05, with time point as a repeated factor. This may be suggestive of evidence for cumulative toxicity if trending in a clinically deleterious direction.

Health-related quality of life, measured by PROMIS-29, is included as a tertiary measure of gross changes in health. PROMIS-29 includes 7 validated subscales (physical function, anxiety, depression, pain intensity, pain interference, sleep, social interactions), each of which will be calculated as a mean by group, and means will be compared between xanthohumol and placebo, with the primary comparison being of mean changes from baseline to week 8. Significance of changes in QOL measures will be tested according to the same plan outlined for laboratory markers, above.

Given the small sample size of this clinical trial, changes in results related to the secondary objective including inflammatory cytokines, gut permeability biomarkers, and markers of endotoxemia are considered preliminary. Thus, our principal assessment for such exploratory outcomes will be of estimated effect sizes, calculated as a *Cohen’s d* statistic for difference between study groups, using change from baseline at each 2-week interval. The primary effect estimate will be for changes from baseline to week 8. We will also perform formal statistical comparisons with adjustments for skew as outlined in our primary objective analysis. The following will be considered evidence of possible effects for our secondary objective: (1) statistically significant effects for changes in cytokines of interest and/or (2) calculation of a Cohen’s *d* value for effect size estimation greater than 0.5 (with larger *d* indicating a larger potential effect size).

Finally, respective of our tertiary objective, we will identify and quantify xanthohumol and xanthohumol-derived metabolites in plasma, urine, and stool samples. The metabolite profile data will then be used for correlation with the time-resolved gut microbiome analysis. This approach will allow us to determine links between metabolite profiles, gut microbiome compositions, and proteomic data [29–36]. We will also quantify bile acids in plasma and stool samples as well as develop analytical methods for assessing microbiome compositional changes as appropriate to the study sample size [32, 37–49].

## Oversight and monitoring

### Composition of the coordinating center and trial steering committee {5d}, data monitoring committee, its role and reporting structure {21a, 21b}

This trial is registered with ClinicalTrials.gov (NCT03735420) and includes regulatory oversight by the DSMB, FDA, the National Center for Complementary and Integrative Health (NCCIH), and the Institutional Review Boards of the National University of Natural Medicine (NUNM, approval RB9718). The DSMB will be chaired by Robert Martindale, MD, PhD and Jessica Minnier, PhD, of Oregon Health & Science University along with Robyn Dreilbelbis, DO, of Western University of Health Sciences, to ensure impartial monitoring of safety data including adverse events and laboratory markers. The board will meet on a regular basis, including at half-enrollment, to ensure monitoring of abnormal laboratory values and reporting of serious adverse events is being conducted in a timely manner. The FDA provided review and approval of the study protocol and Data Safety and Monitoring Plan (DSMP) during the Investigational New Drug (IND) application process; the FDA requested clarifications on participation criteria and halting criteria during this review. NCCIH periodically monitors trial progress via regular annual progress reports completed by the study’s Principal Investigators, evaluates progress according to a projected Study Accrual and Retention Plan (SARP), and reports from the DSMB, as well as through review and approval of all protocol modifications prior to implementation.

All study investigators will meet on a regular basis to discuss progress and developments throughout the trial. Minutes for these minutes will be maintained by the Study Coordinator, Ms. Emily Stack, and may be provided to the trial’s external auditing company upon request.

### Adverse event reporting and harms {22}

Adverse event monitoring will occur using a standardized, multi-system, 81-item adverse event questionnaire based on the National Cancer Institute’s Common Terminology Criteria for Adverse Events. The adverse event questionnaire will be administered verbally by study staff at each of the clinical visits, including the initial screening visit, with a query to determine if other symptoms not elicited by the questionnaire have been experienced. Participants will be encouraged to contact study staff between study visits if they are experiencing any new, unusual, or bothersome symptoms. Adverse events reported between study visits (i.e., not reported on the 81-item adverse event interview during scheduled visits) will be termed a “spontaneous report” adverse event and will be documented within the participant’s file by the study staff to capture the severity/grade of the report, any clinical actions taken, and the current state of the adverse event. Following a spontaneous report, the study team, in coordination with a participant’s physician as needed, will develop a response or monitoring plan, which will also be documented.

All reported adverse events, as well as the length of duration, will be reported upon trial completion with a comparison of frequency between groups. Attributability to the intervention will be determined on a case-by-case basis by the clinical investigators and DSMB members according to accounts of the event(s) in previous research, whether the symptom was present at baseline, the severity and clinical trajectory of the symptom (i.e., did it worsen with accumulated exposure), whether similar symptoms accumulated in one group vs. another, and/or if the symptom was subjectively attributed to another cause *by the participant* (e.g., wrist pain following an injury at work).

Severity of adverse events will be classified by the Clinical Investigators as grade 1 through 5 based on the following definitions: grade 1/mild (an event that requires no treatment and does not interfere with the participant’s daily activities), grade 2/moderate (an event that may cause some interference with the participant’s daily activities and/or requires medical intervention which may include over-the-counter medications), grade 3/severe (an event which limits basic self-care and/or requires hospitalization), grade 4/life threatening (an event which results in hospitalization), or grade 5/fatal (death). Adverse events will then be classified as serious or non-serious based on FDA and Federal Food Drug and Cosmetic Act definitions [50]. Adverse events will be considered serious if a participant’s outcome includes the following: a life-threatening experience, inpatient hospitalization, disability or incapacity, death, or a medical or surgical intervention to prevent one of these outcomes. All other adverse events will be designated “non-serious.” No attributable serious adverse events are anticipated during the trial unless a previously unidentified allergy is elicited by the interventional product or excipients.

### Frequency and plans for auditing trial conduct {23}

External, third-party audits will be conducted by Westat Corporation prior to trial initiation, at half-enrollment, and upon trial completion. Regular, weekly meetings between investigators will be conducted with maintained minutes to remain updated on trial changes and developments.

### Plans for communicating important protocol amendments to relevant parties {25}

Any protocol amendments will be sent to NCCIH, the US FDA, and IRBs according to each entity’s respective timeline.

### Dissemination plan {31a, 31c}

The investigation team anticipates publishing trial results through journal publication without any restrictions. Data sharing requests will be considered and require approval from all PIs prior to release.

## Discussion

The main objective of the phase I XMaS trial is to examine the safety and tolerability of daily xanthohumol administration in healthy adults over an 8-week time frame. This objective will be accomplished by monitoring routine clinical laboratory parameters, assessing quality of life, and administering standardized adverse event queries throughout the study and using these data to make a composite assessment of clinical safety, with input from the FDA and NCCIH. Tolerability of xanthohumol will be assessed by monitoring the number and proportion of participants who discontinue the intervention citing adverse effects, changes in the seven health-related quality of life domains captured via the PROMIS-29 questionnaire, and the severity and duration of symptoms, for comparison between treatment and control groups.

Preclinical studies have demonstrated that xanthohumol has multiple therapeutic properties including modulating inflammatory pathways by acting as an FXR agonist and via inhibition of NFκB activation, regulation of gut permeability and bile acid metabolism by acting as an FXR modulator, and regulation of antioxidant protein expression via activation of NRF2 [7–13, 15, 51]. By regulating these transcription factors, xanthohumol may impact expression of numerous downstream genes in vivo and therefore has therapeutic promise for multiple disease states, including Crohn’s disease (Table 1). Furthermore, xanthohumol acts as a prebiotic for intestinal microbiota and, along with its bacterial metabolites, alters the gut microbiota [5]. Therefore, additional aims of the phase I XMaS trial are to collect data on biomarkers related to inflammation, gut permeability, bile acid metabolism, xanthohumol metabolism, and the impact of xanthohumol on gut microbiota to assess if these mechanisms are modified in vivo.

The design of the phase I XMaS trial has both strengths and limitations. Strengths include a triple-masked, randomized, placebo-controlled trial designed to minimize bias and optimize causal inference. The robust trial design includes a thorough, standardized method for monitoring the safety and tolerability. Additional strengths include collaboration amongst clinical and laboratory scientists with expertise in human studies of natural products, preclinical and translational xanthohumol research, metabolomics, and metagenomic sequencing. A limitation of this trial is the evaluation of a single dosage (24 mg per day) of xanthohumol over an 8-week time frame, rather than escalating the dose as can be done in phase I trials. Therefore, it is possible that the dosage provided may be subtherapeutic, insufficient for determining potential toxicity of xanthohumol, and/or insufficient to demonstrate mechanistic activity in vivo. However, considering the dose administered is approximately 20–100-fold the typical daily dietary intake (depending on type and quantity of beer consumption), we maintain the chosen dose remains sufficiently large to elicit a measurable effect should one exist. Similarly, but in the opposite direction, it is possible xanthohumol exerts a hormetic effect, in which case the chosen dose may greatly exceed an effective dose (although toxicity would likely be detected in this case) [52]. Another limitation is the lack of data collection on customary dietary intakes and patterns in participants, which may impact our ability to interpret the impact on the microbiota and metabolomics, especially if uniform and specific dietary patterns are not maintained across participants.

Should preclinically-defined mechanisms of xanthohumol be supported by the results of the phase I XMaS trial, the results will inform future translational trials that examine its safety, efficacy, and biologic mechanisms of xanthohumol in clinical populations. Results of this work have the potential to yield numerous original biomedical contributions that may be informative to key stakeholders, including grant-funding bodies, regulatory bodies, clinical and basic science researchers, industry, practicing clinicians, patients, and consumers.

## Trial status

IRB #: RB9718. Approval date: September 13, 2018. Protocol version 1.0 November 21, 2019. Protocol version 1.8

Recruitment start date: August 15, 2019

Projected recruitment end date: April 1, 2020

## Supplementary information

**Additional file 1.** Participant informed consent

## Abbreviations

FXR: Farnesoid X receptor
TNF-α: Tumor necrosis factor-alpha
IFN-ɣ: Interferon-gamma
NFκB: Nuclear factor-kappa B
BMI: Body mass index
AST: Aspartate aminotransferase
ALT: Alanine aminotransferase
GGT: ɣ-Glutamyl transferase
eGFR: Estimated glomerular filtration rate
BUN: Blood urea nitrogen
LPS: Lipopolysaccharide
LBP: Lipopolysaccharide-binding protein
NUNM: National University of Natural Medicine
OSU: Oregon State University
PNNL: Pacific Northwest National Laboratory
DSMB: Data Safety Monitoring Board
CRFs: Case report forms
CI: Confidence interval
