# Correction to: Xanthohumol microbiome and signature in healthy adults (the XMaS trial): a phase I triple-masked, placebo-controlled clinical trial

**DOI:** 10.1186/s13063-020-04834-w

**Published:** 2020-10-26

**Authors:** Ryan Bradley, Blake O. Langley, Jennifer J. Ryan, John Phipps, Douglas A. Hanes, Emily Stack, Janet K. Jansson, Thomas O. Metz, Jan Frederik Stevens

**Affiliations:** 1 National University of Natural Medicine, Portland, USA; 2 Pacific Northwest National Laboratory, Richland, USA; 3 Oregon State University, Corvallis, USA

**Correction to: Trials (2020) 21:835**

**https://doi.org/10.1186/s13063-020-04769-2**

Following publication of the original article [1], we were notified that in the first paragraph of the Interventions section (line 202), “Participants randomized to placebo will be administered 288 mg of rice protein, 133.4 mg microcrystalline cellulose” should read “Participants randomized to placebo will be administered 288 mg of rice protein,109.3 mg microcrystalline cellulose”.

The original article has been corrected.
