# Supplementary material for: Xanthohumol microbiome and signature in healthy adults (the XMaS trial): a phase I triple-masked, placebo-controlled clinical trial
Source: Trials. 2020 Oct 7;21:835. doi: 10.1186/s13063-020-04769-2 (PMC7542976; doi:10.1186/s13063-020-04769-2)
Supplement: Supplementary file 1 — Additional file 1. Participant informed consent [file 13063_2020_4769_MOESM1_ESM.pdf]

Participant Informed Consent

Title of Study: Natural Product Signature in Healthy Adults

**Principal Investigator:**	Ryan Bradley, ND, MPH	503-552-1862
**Clinical Investigator:**	Ryan Bradley, ND, MPH	503-552-1862
	Jennifer Ryan, ND, MS	503-552-1744
	Blake Langley, ND	
**Co-Investigators:**	Jennifer Ryan, ND, MS	503-552-1744
	John Phipps, PhD	503-552-1773
**Study Coordinators:**	Emily Stack	503-552-1777
	Lita Buttolph, PhD	503-552-1746
**Biostatistician:**	Doug Hanes, PhD	503-552-1741
**Post-doctoral fellow:**	Blake Langley, ND	
**Research Assistants:**	Sara Guedry	
	Tyler Weed	

Sponsor: National Center for Complementary and Integrative Health (NCCIH)

The National University of Natural Medicine's Helfgott Research Institute is conducting a research study to determine how a natural product derived from the Hops plant is metabolized by the bacteria in the gut, i.e., the microbiota. The study is funded by National Institutes of Health, National Center for Complementary and Integrative Health (NCCIH).

INTRODUCTION

The National University of Natural Medicine (NUNM) is conducting a research study, in the form of a clinical trial, to assess how a natural product derived from the Hops plant is broken down by the bacteria in the intestines. You are being asked to be in this research study**.** This form provides you with information, reviews your rights, and explains your role in this study, should you decide to participate. A member of the research team will describe this study to you and answer all of your questions. Please read the information below and ask questions about anything you don't understand before deciding whether or not to take part in this study. We will give you a copy of this form for your records.

WHAT IS THE STUDY ABOUT?
The purpose of this study is to determine how a natural product derived from the Hops plant impacts and is broken down by the bacteria in the intestines. Results gained from your participation will be used to determine the safety profile, as well as to serve as a control (comparison) group for future studies in adults with chronic disease(s).

To be eligible to participate in the study, you must be:
Inclusion criteria

- An adult aged 21-50 years
- Willing to take a placebo or experimental natural product for 8 weeks
- Willing to have blood drawn every other week, and fast for 10-12 hours before these visits
- Willing and able to collect stool samples at home every other week before each study visit
- Willing and able to collect a 24-hour urine sample every other week before each study visit
- Able to speak, read and understand English
- Must be able to provide written informed consent
- Non-smokers (including tobacco and Cannabis products, combusted or vaporized)
- For women of child-bearing potential, willingness to use an IUD or two other concurrent forms of birth control (e.g., 2 of the following categories: condoms, spermicide-containing gels, films or sponges; and/or vaginal rings) to prevent pregnancy while enrolled.

You will not be eligible to participate if:
Exclusion criteria

- History of any chronic disease including, but not limited to: diabetes (type 1 or 2); uncontrolled hypertension; coronary artery disease resulting in angina; cardiovascular disease requiring PCI, bypass, or past myocardial infarction or stroke; blood disease including current anemia; cancer (except non-melanoma skin cancer) within the last year or still requiring chemotherapy or hormonal therapy; chronic kidney disease; liver disease including viral hepatitis, non-alcoholic fatty liver disease, or alcoholic hepatitis/cirrhosis; any immunocompromising condition including HIV/AIDs or organ transplant requiring anti-rejection medications; chronic osteoarthritis requiring joint replacement or daily use of NSAIDs; chronic endocrine condition including but not limited to: Cushing's, Addison's, Hashimoto's thyroiditis, Grave's disease, etc.
- Body Mass Index (BMI) less than 20 or greater than 30
- Consumption of more than 1 beer per day. Candidates will be given the option to stabilize intake to this level for 14 days and re-contact the study team.
- Use of NSAIDs more than once per week for headaches, routine aches/pains, etc.
- Use of any prescription drugs, including oral contraceptives (due to potential interference with mechanisms under investigation)
- Use of prescription opioids for any reason within the past 3 months
- Use of prescription corticosteroids for any reason within the past 3 months

- Free of acute viral or bacterial infection, or recent infection within the last 14 days or still requiring prescription medication for treatment
- Free of recent acute trauma occurring within the last 14 days
- Currently or recent (within last 14 days) taking any dietary supplements containing herbal "anti-inflammatory" ingredients including: flavonoids, curcumin, turmeric, fenugreek, hops, rosemary, ginger, white willow, Devil's claw, fish oil (doses>1g/day), quercetin, or isolated compounds from the above. Candidates will be given the option to "wash out" for 14 days and re-contact the study team.
- Currently receiving intravenous nutrition support therapy (or within the last 30 days)
- Currently taking anti-coagulant or anti-platelet prescription medications (or they were taken within the last 30 days)
- Currently taking antibiotic, antiparasitic, or antifungal medications orally or intravenously (or they were taken within the last 30 days)
- Initiation of or changes to supplements or medications within 14 days prior to screening. Candidates will be given the option to stabilize their allowable dietary supplements and medications for 14 days and re-contact the study team
- Initiation of or changes to an exercise regimen within 14 days prior to screening. Candidates will be given the option to stabilize their exercise for 14 days and re-contact the study team.
- Initiation of or changes to a food plan within 14 days prior to screening. Candidates will be given the option to stabilize their diet for 14 days and re-contact the study team.
- Current involvement or within 30 days prior to screening of a significant diet or weight loss program, such as NutriSystem, Jenny Craig, Atkin's or other low-carb diet programs, or very low calorie liquid diet programs (such as Optifast, Medifast, and/or HMR)
- Hospitalization (for any reason other than an elective medical procedure) within 3 months prior to screening
- Gastrointestinal surgery within 3 months prior to screening
- Undergoing UV therapy (e.g. treatment for skin conditions such as psoriasis).
- Engaging in vigorous exercise (e.g. activity at or above 80% of maximum perceived rate of exertion) more than 6 hours per week.
- Women who are lactating, pregnant or planning pregnancy within the next four months
- Typical intake of more than 2 alcohol-containing beverages per day, more than 14 per week, or more than 4 in any single day within the past 14 days. Candidates will be given the option to stabilize intake to these levels for 14 days and re-contact the study team.
- Smoking tobacco and nicotine products (combusted or vaporized)
- Use of illicit drugs/substances (such as but not limited to cocaine, phencyclidine [PCP], and methamphetamine) within 30 days of screening
- Use of inhaled or ingested *Cannabis* products, including CBD. Candidates will be given the option to discontinue intake for 14 days and re-contact the study team.
- Currently participating in another interventional research study or participated in another interventional study within 30 days of screening

HOW LONG WILL I BE IN THE STUDY?
You will be involved in this study for 8 weeks (about 2 months) after you begin taking the study product.

WHAT WILL HAPPEN IN THE STUDY?
If you are eligible, you will be asked to take the study product once per day with your first daily meal for 8 weeks. You will attend seven in-person study visits. You will complete symptom and quality of life questionnaires.

Your blood will be drawn at each of the study visits, excluding the randomization visit, and you will also be asked to collect a stool sample and a 24-hour urine sample at home using a provided kit before each visit.

The seven in-person study visits will be conducted at the NUNM Helfgott Research Institute in Portland, OR. The specific tests and procedures that will take place at each visit are described below.

<u>Baseline Visit (Week 0)</u>
During the baseline visit:

- We will ask you to sign this consent form after you have read it and had any questions or concerns addressed
- We will ask you to complete forms about your medical symptoms
- One fasting blood draw to collect 7 tubes of blood
- At each visit we will store a small volume (7ml) of the collected blood and urine for the purposes of potential future analyses if you sign the optional consent below
- We will provide you with a stool and urine sample collection kit and request you return the two samples the following day
  Estimated time to complete: 45 minutes

<u>Randomization Visit (day after Baseline Visit)</u>
During the randomization visit:

- Once we have received your stool and urine sample collection we will assign you to one of the two groups randomly like by the flip of a coin. Neither you nor the study coordinator will be able to choose which group you are assigned
- We will provide a supply of the study supplement and instructions on how to take them
- We will provide a urine and stool collection kit for your next visit
  Estimated time to complete: 15 minutes

<u>Bi-weekly Clinical Visit (Week 2)</u>

During the week 2 visit:

- We will ask you to complete forms about your medical symptoms
- One fasting blood draw to collect 5 tubes of blood
- We will ask you to provide your stool and urine sample that was collected that morning
- We will provide a urine and stool collection kit for your next visit
- Participants will be asked to return any unused product at their next visit
  Estimated time to complete: 60 minutes

Midpoint Clinical Visit (Week 4)

During the midpoint visit:

- We will ask you to complete forms about your medical symptoms
- We will collect any unused supplements
- One fasting blood draw to collect 7 tubes of blood
- We will ask you to provide your stool and urine sample that was collected that morning
- We will provide a supply of the study supplement and instructions on how to take them
- We will provide a urine and stool collection kit for your next visit
  Estimated time to complete: 60 minutes

Bi-weekly Clinical Visit (Week 6)

During the week 6 visit:

- We will ask you to complete forms about your medical symptoms
- One fasting blood draw to collect 5 tubes of blood
- We will ask you to provide your stool and urine sample that was collected that morning
- We will provide a urine and stool collection kit for your next visit
- Participants will be asked to return any unused product at their next visit
  Estimated time to complete: 60 minutes

Trial Closeout- Week 8

During the trial closeout visit:

- We will ask you to complete forms about your medical symptoms
- We will collect any unused supplements
- One fasting blood draw to collect 7 tubes of blood
- We will ask you to provide your stool and urine sample that was collected that morning
  Estimated time to complete: 60 minutes

	**Phone Screening**	**Screening Visit**	**Baseline Visit- Week 0**	**Randomization Visit – day after baseline**	**Bi-weekly Visits (Weeks 2,6)**	**Midpoint Visit- Week 4**	**Trial Closeout- Week 8**

Oral waiver of consent	X						
Initial eligibility screening	X						
Written informed consent		X	X				
W9 Form		X					
Group assignment				X			
Health interview		X	X		X	X	X
Lab studies		X	X		X	X	X
Blood collection		X	X		X	X	X
Urine collection				X	X	X	X
Stool collection				X	X	X	X
Product dispensed				X		X	
Stipend provided						X	X
Participant Discharged							X

WHAT IF I HAVE QUESTIONS?

Any participant with questions or concerns about the study may contact the Study Coordinators, Emily Stack, at 503-552-1777 or Lita Buttolph, at 503-552-1746. You may also contact Dr. Ryan Bradley at 503-552-1862.

DO I HAVE TO BE IN THE STUDY?

It is up to you to decide if you want to be in the study. This study is completely voluntary even if you are a patient at NUNM Clinic or are an NUNM student or employee. Choosing not to take part in the study or choosing to leave the study will not affect your relationship with the Helfgott Research Institute, NUNM Clinic, NUNM, or your ability to participate in future research with NUNM.

If you are eligible and wish to join the study, you must sign this Consent form. If you do not sign the Consent form you cannot join the study.

WHAT IF I DON'T WANT TO BE IN THE STUDY?

You can choose at any time to not join the study or to leave the study, even if you are affiliated with NUNM. You can also choose to stop participating in the study without giving a reason why.

ARE THERE ANY COSTS?
There are no costs for you to be in this study. All study-related tests and supplements are free.

WILL I BE PAID FOR BEING IN THE STUDY?
If you are eligible and enroll in the study, you could be compensated with up to $550 for the inconvenience of collecting your urine and stool, and attending the scheduled clinical visits. Participants will receive $50.00 for the completion of the Screening Clinical Visit, $100.00 for each of the completed Baseline Clinical Visit and Bi-Weekly Clinical Visits. A check will be mailed to participants by the NUNM business office within six to eight weeks after each participant has completed the study (or after their participation has ended if they do not complete the entire study).

ARE THERE ANY RISKS?
Risks associated with any dietary supplement include gastrointestinal symptoms such as gas, bloating, digestive upset and change in bowel movement frequency or consistency. Also there is a chance of identifying a previously unknown allergy, and therefore a risk of rash, breathing difficulty and serious allergic reaction.

We will ask you about symptoms at each study visit and any side effects will be recorded. We may ask you to discontinue the study product if we believe it would be in your best interest.
There will be six blood draws during this study. There is a small chance that the needle will cause some discomfort, bruising, redness or bleeding at the needle puncture site. Occasionally, individuals may feel lightheaded or faint during or immediately after a blood sample is collected. There is a rare risk of swelling around the vein or of infection.

We will do everything we can to keep your records private, yet there is always a small risk of a breach of confidentiality of personal health information. These risks have been addressed and minimized as much as is possible by keeping data in locked cabinets and on password protected and encrypted computers, and by using ID numbers instead of names on all data collected.

There is a chance that some participants may confuse research study visits for routine clinical care. We would like to emphasize that all study visits are for research purposes only and not for medical care.

There could be risks that we are unaware of at the time of the study. You will be told about any new information that may affect your willingness to participate in the study.

Participant's Initials: __________

If you experience any side effects while participating in the study or within seven (7) days after the study ends, please contact Emily Stack at 503-552-1777 or Lita Buttolph at 503-552-1746 as soon as possible. If your study visits are being conducted at NUNM's Helfgott Research Institute in Portland, OR, you may also contact Dr. Ryan Bradley at 503-552-1862.

WHAT IF I FEEL I'VE BEEN HURT BY TAKING PART IN THE STUDY?
If you feel you have been injured or harmed by taking part in this study, please contact Dr. Ryan Bradley, ND, MPH at (503) 552-1862. If you feel you were harmed while taking part in this study, you may be treated at NUNM Clinic in Portland, OR. However, NUNM and the NUNM Health Center do not offer to pay the cost of this treatment.

If you feel your rights have been violated or you have been harmed by this study, please contact the NUNM Institutional Review Board at 503-552-1758.

ARE THERE ANY BENEFITS?
There may be little direct benefit other than the personal satisfaction of contributing to health research and community. The knowledge gained from the proposed research has the potential to directly inform the development of new therapeutic agents for the treatment of inflammatory bowel disease, including drugs and medical food products.

YOUR PRIVACY IS IMPORTANT
The Health Insurance Portability and Accountability Act (HIPAA) is a federal law to protect your privacy. Protecting your privacy is very important to us.

During this study we will you ask about your medical history, we will do this with questionnaires, medical exams, blood draws, etc. This information will be used to provide data for the study. Your personal health information will be kept private and only authorized study staff will have access to this information. We will use a de-identified study number instead of your name. All paper forms will be kept in a locked, secure office. All electronic data will be stored on password-protected computers. Your name will not be used in any publications or presentations about this study.

During the study, you may not be given access to medical information about you that is part of the study. When the study is over, you may request certain medical information collected about you that is part of your study medical record.

Any identifying personal health information about you will not be shared with anyone other than authorized study staff at NUNM.

By signing this consent form you are stating that we can use your health information in the ways mentioned above for this study. You are not waiving any of your legal rights by signing this form.

You have the right to take away your permission to use your health information and any blood samples collected as part of the study. In order to do so you must send a written request to:

Dr. Ryan Bradley, ND, MPH
Helfgott Research Institute
2220 SW 1st Avenue,
Portland, Oregon 97201

Once your letter is received no additional information about you or blood samples will be collected from you for this study. Any data collected before we receive your letter will continue to be used for the study. Taking away your permission to use your health information will not affect your relationship with NUNM.

We are collecting only the personal health information that we need for the specific purpose of this study. Your personal health information cannot be used for additional research purposes.

NUNM may be required to provide copies of your personal information to Federal or other government agencies such as the Food and Drug Administration (FDA) as required by law. It may also be required to provide copies to the Institutional Review Board (IRB) or other groups that monitor the safety and welfare of study participants.

If your personal health information is disclosed by this authorization to an individual or agency not covered by laws that prohibit re-disclosure, your personal health information may not remain confidential. However, Oregon law does not allow re-disclosure of HIV/AIDS information, mental health information, genetic information, and drug/alcohol diagnosis, treatment, or referral information.

Your permission to use your identifiable health information (your HIPAA authorization) will expire when the study is complete.

Signatures:

By signing this consent form it means the following:

- I have read this form and understand it.
- I know my rights have not been waived by signing.
- I have had all of my questions answered and I know whom to ask if I have more questions.
- I want to join the study.
- I know I can leave the study at any time and do not have to give a reason.

____ _____________
Signature of Participant *Date*

____
Printed name of participant

____ _____________
Signature of Consenter *Date*

____
Printed Name of Consenter

Thank you for participating in our research study!

Consent for Subsequent Re-Analysis of Stored Samples

From time to time new information emerges and there may be a scientific need to measure additional factors in your blood or urine to help us better understand the results of the main study, or to pursue new scientific questions that were not known at the time of the main study. With your consent, the samples collected from you in this trial will be used for subsequent analyses for related studies, should they become necessary. A small volume of blood and urine will be stored and frozen at -70C for the purposes of potential future analyses.

I hereby consent that all samples collected from this trial can be used to measure:

- **________ (initial) Blood-based biomarkers including routine labs and experimental measures**
- **________ (initial) Urine-based biomarkers including routine labs and experimental measures**

____ _____________

Signature of Participant *Date*

____

Printed name of participant

- ________ *(Initial) I do not give consent to store additional Blood or Urine samples*

Thank you for participating in our research study!
